# Vitamin C Deficiency in a 24-Year-Old Male Resulting in Anemia From Hematuria and Impaired Erythropoiesis Requiring Multiple Transfusions

**DOI:** 10.7759/cureus.87592

**Published:** 2025-07-09

**Authors:** Vinh Dao, Katherine Kessler, Tark Abou-Elmagd, Ashish Subedi, Tariq Nazir

**Affiliations:** 1 Internal Medicine, Cape Fear Valley Health, Fayetteville, USA; 2 Psychiatry, Cape Fear Valley Health, Fayetteville, USA; 3 Hematology and Medical Oncology, Cape Fear Valley Health, Fayetteville, USA

**Keywords:** anemia, autism spectrum disorder (asd), impaired erythropoiesis, malnutrition, packed red blood cell transfusion, vitamin c deficiency

## Abstract

Vitamin C deficiency, or scurvy, is an often-overlooked nutritional disorder in modern clinical practice, particularly in high-income countries, such as the United States. It is most commonly associated with food insecurity, poverty, and generalized malnutrition. However, this case underscores an equally important yet less recognized risk factor: autism spectrum disorder (ASD). Individuals with ASD are at high risk given their sensory sensitivities and food selectivity, and frequently exhibit restricted dietary patterns that limit their nutritional intake.

We present the case of a 24-year-old male with a known diagnosis of ASD who was admitted with symptomatic anemia, hematuria, spontaneous bruising, and mucosal bleeding. Initial workup revealed severe normocytic anemia with poor reticulocyte response and evidence of renal dysfunction, necessitating multiple transfusions. Physical examination findings, which included perifollicular hemorrhage and corkscrew hairs, and a detailed dietary history revealed a highly restricted intake. His serum vitamin C level was subsequently found to be undetectable. After several days of vitamin C supplementation and multiple transfusions, the patient showed significant clinical improvement, with resolution of hematuria and normalization of hemoglobin levels on follow-up.

This case illustrates the diagnostic challenges posed by micronutrient deficiencies presenting with nonspecific and severe clinical manifestations, such as anemia, hematuria, and renal dysfunction. It also reinforces the importance of considering atypical etiologies in patients with developmental disorders, particularly those with restrictive eating behaviors. Early dietary assessment and nutritional screening should be integral components of care for patients with ASD, especially when clinical presentations are unexplained or involve bleeding, bruising, or fatigue.

## Introduction

Vitamin C, or ascorbic acid, is a water-soluble essential micronutrient that plays a key role in collagen synthesis, iron absorption, neurotransmitter biosynthesis, and immune function. A deficiency in vitamin C can lead to a well-documented but often overlooked condition known as scurvy. Vitamin C deficiency can present with a variety of symptoms. It can present as nonspecific symptoms ranging from fatigue and malaise to severe anemia, spontaneous bleeding, and impaired wound healing. While often considered a historical disease, recent data estimates the prevalence of vitamin C deficiency to be approximately 7.1% in the United States, highlighting its continued relevance in clinical practice [1].

Though commonly associated with individuals experiencing food insecurity, homelessness, substance use disorders, or eating disorders, vitamin C deficiency can also affect individuals without obvious signs of malnutrition. One such at-risk population includes individuals with autism spectrum disorder (ASD). These individuals frequently have rigid food preferences and aversions to specific textures, tastes, and appearances. These sensory sensitivities can significantly restrict dietary variety, often leading to inadequate intake of key nutrients, including vitamin C [2]. This can result in the avoidance of certain fruits and vegetables, making these individuals higher risk for having nutritional deficiencies.

The nutritional risks associated with ASD are frequently underrecognized in adult populations, as much of the literature and clinical focus tends to center on children. However, dietary rigidity can persist into adulthood, making it imperative for healthcare providers to maintain awareness of nutritional gaps in patients with ASD across all age groups. Complicating matters further, the symptoms of vitamin C deficiency can mimic other more common diagnoses such as urinary tract infections, glomerulonephritis, or hematologic disorders, making timely diagnosis especially challenging in the absence of classic signs such as gingival bleeding or corkscrew hairs.

In this case report, we present a young adult male with ASD who developed profound anemia and gross hematuria secondary to severe vitamin C deficiency secondary to his severely restricted diet. This case highlights the diagnostic complexity of micronutrient deficiencies and the importance of maintaining a high index of suspicion in patients with ASD who present with nonspecific symptoms and dietary limitations. Additionally, it underscores the need for routine nutritional assessments in at-risk populations and the value of multidisciplinary care to prevent severe nutritional deficiencies and their life-threatening complications.

## Case presentation

A 24-year-old male with a past medical history of ASD, asthma, hypertension, and obesity presented to his primary care physician for concerns of spontaneous bruising along his bilateral lower extremities, which had been ongoing for two weeks. He noted having a history of spontaneous bruising on his bilateral hands for four years that continued to spontaneously resolve. He reported having normal laboratory workups during these episodes. He also reported having one week of dark-colored urine that was described as cola colored. He also reported having epistaxis and gum bleeding while brushing his teeth.

An outpatient CBC was shown to have a hemoglobin (Hgb) of 5.0, which prompted the patient to go to the emergency department (ED). In the ED, vital signs were notable for a heart rate of 121 and BP 108/69. Physical exam was notable for perifollicular hemorrhage, corkscrew hairs, and no active bleeding. Repeat CBC demonstrated an Hgb of 5.0 and hematocrit of 14.9. The percentage of reticulocytes was 0.6, and the red blood cell count was 2.4 × 10^6^ µL, with a reticulocyte production index of 0.1. This indicated that the bone marrow had an inadequate response to the patient's anemia. Basic metabolic panel (BMP) was notable for creatinine (Cr) 3.26 and blood urea nitrogen (BUN) 35, which were both previously within normal limits. Total bilirubin was 1.1. The protein/Cr ratio was 1513. Iron panel, prothrombin time (PT), international normalized ratio (INR), vitamin B12, folate, lactate dehydrogenase (LDH), and haptoglobin were within normal limits. Urinalysis was notable for 2+ protein and 3+ blood. Peripheral smear was negative for any blasts or schistocytes. Laboratory results are summarized in Table 1.

**Table 1:** Laboratory results BUN, blood urea nitrogen; PT, prothrombin time; INR, international normalized ratio; LDH, lactate dehydrogenase

Laboratory Test	Value	Reference Range
Hemoglobin	5	13.5-18 g/dL
Hematocrit	14.9	40.5-50.4%
Red Blood Cell Count	2.4	×10^6^/uL
Reticulocyte Percentage	0.6	0.6-2.3%
Platelets	375	150-450 × 10^3^/uL
Total Bilirubin	1.1	0.3-1.0 mg/dL
BUN	35	7-25 mg/dL
Creatinine	3.26	0.6-1.3 mg/dL
Urine Protein/Creatinine Ratio	1513	0-200 mg/g creatinine
Iron	46	50-212 μg/dL
Ferritin	91.8	23.9-336.2 ng/mL
PT	12.1	9.7-12.4 seconds
INR	1.1	0.9-1.1
Vitamin B12	471	180-914 pg/mL
Folate	11.7	≥5.0 ng/mL
LDH	244	137-275 U/L
Haptoglobin	213.9	44-215 mg/dL
Complement C3	128	>41 U/mL
C4 Complement	21	12-38 mg/dL
Vitamin C	<0.1	0.4-2.0 mg/dL

The patient was admitted and was transfused with two units of packed red blood cells (PRBCs) with subsequent improvement in Hgb to 7.1. The following day, his Hgb again fell to 6.7, resulting in him receiving another transfusion. The patient also reported having increased dark urine overnight that decreased throughout the day. Paroxysmal nocturnal hemoglobinuria flow cytometry was sent for further evaluation. Further labs, including direct antiglobulin test, complement levels, parvovirus, and glomerulonephritis labs, were negative. With the patient's normal LDH, haptoglobin, borderline normal bilirubin, lack of schistocytes, and negative direct antiglobulin test (DAT), hemolysis was ruled out. Regarding his proteinuria, his complement levels were within normal limits, and the glomerulonephritis labs were negative. 

Given the patient’s physical exam findings, more history was obtained regarding his diet. His diet was limited due to his severe texture aversion from his ASD and mostly consisted of cheeseburgers, quesadillas, and fried foods. Vitamin D was seven. A vitamin C lab was ordered, but was not available in-house. While waiting for vitamin C levels, the patient continued to have hematuria and was transfused a total of five units throughout his hospitalization. Eventually, vitamin C levels came back to <0.1.

**Figure 1:**
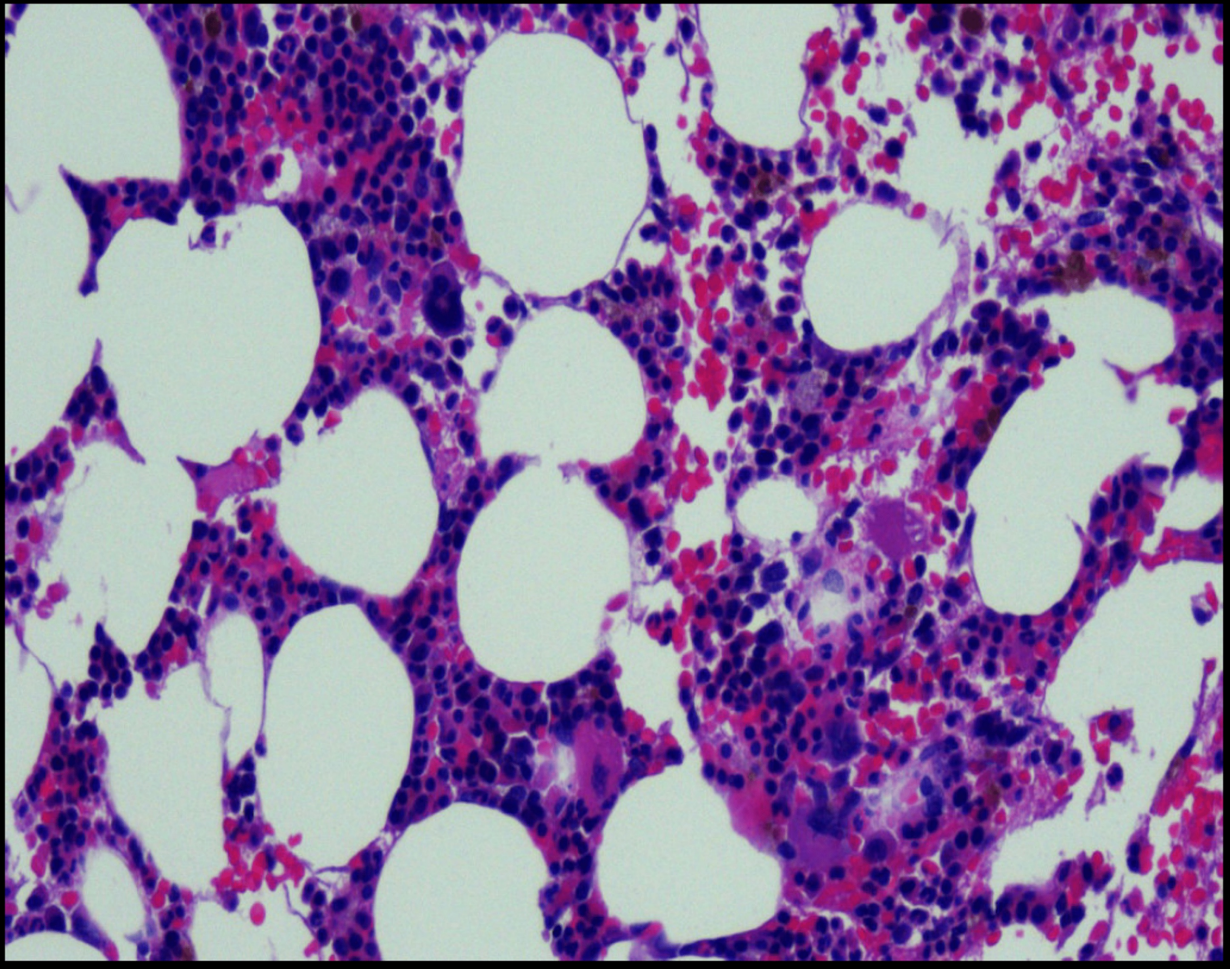
Bone marrow biopsy with variably cellular marrow with overall mild hypocellularity for age (approximately 40-60% cellularity), with trilineage hematopoiesis showing a decreased myeloid to erythroid ratio with no evidence of myelodysplasia or other neoplasia

A bone marrow biopsy was also done, given his poor bone marrow response. His bone marrow biopsy showed mild hypocellularity with a decreased myeloid-to-erythroid ratio (Figure 1). Cytogenetics did not reveal any abnormalities.

In addition to his blood transfusion, the patient received multiple days of IV fluids with improvement in his Cr. He also received multiple days of multivitamins, which eventually led to improvement in his hematuria. He was discharged with vitamin C and D supplements and a hematology referral. Approximately two weeks after discharge, the patient was re-evaluated by hematology with improvement in his Hgb to 11.3.

## Discussion

Vitamin C plays a vital role in collagen synthesis, endothelial integrity, and wound healing. In particular, vitamin C is utilized in the synthesis and deposition of type IV collagen, stimulation of endothelial proliferation, and alteration of blood flow through nitric oxide [3]. Vitamin C is also a cofactor for prolyl and lysyl hydroxylase, which catalyzes the hydroxylation of hypoxia-inducible factor-1α and procollagen. This leads to the stabilization of collagen [4]. Inadequate vitamin C levels can result in increased permeability of blood vessels as well as a decrease in their structural function, causing blood seepage into tissue and microvascular bleeding [5]. The change in vascular permeability resulted in our patient’s severe hematuria, requiring him to receive multiple transfusions, as well as his gingival bleeding and easy bruising. With multiple days of vitamin C supplementation, the volume of his hematuria started to decrease and eventually subsided once his vascular integrity was corrected. 

In this case report, we highlighted the importance of considering vitamin C deficiency in evaluating severe anemia. Vitamin C deficiency is often underdiagnosed, as its symptoms can mimic more commonly suspected conditions such as urinary tract infections, glomerulonephritis, or hematologic disorders. Vitamin C deficiency can be prevalent in patients with malnutrition, and it is important to consider this diagnosis in patients with comorbid conditions such as ASD, where restrictive eating patterns are common. Restricted and repetitive behaviors or interests are a key feature of ASD, and this can lead to stringent routines surrounding meals, hypersensitivity to food textures, and an overall limited variability in one’s diet. Commonly cited factors that influence food selectivity include texture, appearance, taste, smell, and temperature. Food selectivity is a challenge amongst ASD patients and can easily lead to inadequate nutrition and numerous micronutrient deficiencies, including vitamin C deficiency [6]. Although nutritional supplementation may address specific micronutrient deficiencies once they are identified, this fails to solve the underlying issue regarding food selectivity and rigid mealtime routines. Applied Behavior Analysis (ABA) is historically the first-line intervention for the treatment of feeding difficulties in patients with ASD and focuses on behaviors, stress, and fixations surrounding diet. However, therapies that focus on targeting sensorial processing have also proven to be beneficial [7]. Multiple evidence-based models exist, including the Sequential Oral Sensory Approach, which is a multi-week interventional program that aims to increase the variety and volume of foods children with ASD can tolerate through desensitization training (focusing on factors such as visual tolerance, interaction, smell, touch, taste, and nutrition) [8]. The difficulty in diagnosis extends even further, as micronutrient deficiencies can be present even in patients who are not underweight [9].

Given the increased risk of micronutrient deficiencies in patients with ASD, routine nutritional screening should be incorporated into standard clinical assessments for individuals with ASD. Physicians and healthcare providers should keep a high level of suspicion for nutritional deficiencies when evaluating patients with unexplained bleeding, anemia, or other nonspecific symptoms. Beyond clinical management, this case underscores the need for a multidisciplinary approach to addressing dietary limitations in individuals with ASD. Primary care physicians, dietitians, behavioral therapists, and psychiatrists should work collaboratively to ensure early detection and intervention. Behavioral feeding therapies, dietary counseling, and supplementation strategies should be considered to prevent severe nutritional deficiencies and their complications.

## Conclusions

This case underscores the importance of considering vitamin C deficiency in patients with atypical presentations of anemia, bleeding, or renal abnormalities, especially in those with ASD. Patients with ASD are at higher risk of developing malnutrition due to their highly restrictive diets secondary to their sensory sensitivities. While vitamin C deficiency is often thought to be rare in developed countries, it remains clinically relevant and can lead to severe, multisystem complications if left unrecognized. Early dietary assessment and a high index of suspicion are critical in at-risk populations, even in the absence of traditional markers of malnutrition. Ultimately, this case highlights the need for multidisciplinary collaboration between primary care, nutrition, behavioral health, and hematology to identify and address nutritional deficiencies before they result in significant morbidity.
